# Genotype–Phenotype Correlations in Angelman Syndrome

**DOI:** 10.3390/genes12070987

**Published:** 2021-06-28

**Authors:** Lili Yang, Xiaoli Shu, Shujiong Mao, Yi Wang, Xiaonan Du, Chaochun Zou

**Affiliations:** 1 Department of Genetics and Metabolism, Children’s Hospital, Zhejiang University School of Medicine, National Clinical Research Center for Child Health, Hangzhou 310052, China; wjpch1@zju.edu.cn; 2 Department of Laboratory Center, Children’s Hospital, Zhejiang University School of Medicine, National Clinical Research Center for Child Health, Hangzhou 310052, China; shuxiaoli2014@zju.edu.cn; 3 Division of Neonatology, Department of Pediatrics, Hangzhou First People’s Hospital, Zhejiang University School of Medicine, Hangzhou 310052, China; maoshujiong@126.com; 4 Department of Neurology, Children’s Hospital of Fudan University, Shanghai 201102, China; yiwang@shmu.edu.cn (Y.W.); duxiaonan3209@163.com (X.D.); 5 Department of Endocrinology, Children’s Hospital, Zhejiang University School of Medicine, National Clinical Research Center for Child Health, Hangzhou 310052, China

**Keywords:** Angelman syndrome, genotype, phenotype, imprinting, intellectual disability, neurodevelopment

## Abstract

Angelman syndrome (AS) is a rare neurodevelopmental disease that is caused by the loss of function of the maternal copy of ubiquitin–protein ligase E3A (*UBE3A*) on the chromosome 15q11–13 region. AS is characterized by global developmental delay, severe intellectual disability, lack of speech, happy disposition, ataxia, epilepsy, and distinct behavioral profile. There are four molecular mechanisms of etiology: maternal deletion of chromosome 15q11–q13, paternal uniparental disomy of chromosome 15q11–q13, imprinting defects, and maternally inherited UBE3A mutations. Different genetic types may show different phenotypes in performance, seizure, behavior, sleep, and other aspects. AS caused by maternal deletion of 15q11–13 appears to have worse development, cognitive skills, albinism, ataxia, and more autistic features than those of other genotypes. Children with a *UBE3A* mutation have less severe phenotypes and a nearly normal development quotient. In this review, we proposed to review genotype–phenotype correlations based on different genotypes. Understanding the pathophysiology of the different genotypes and the genotype–phenotype correlations will offer an opportunity for individualized treatment and genetic counseling. Genotype–phenotype correlations based on larger data should be carried out for identifying new treatment modalities.

## 1. Introduction

Angelman syndrome (AS, OMIM #105830) is an incurable neurodevelopmental disease caused by the loss of function of the maternal copy of ubiquitin–protein ligase E3A (*UBE3A*) and other genes on chromosome 15q11–13 region [1,2]. AS was first described in 1965 by Harry Angelman following a study of three children with similar symptoms [3,4]. During the past decades, our understanding of AS’s clinical phenotypes and genetic pathology has improved. As a genomic imprinting disorder, AS has featured presentations that include global developmental delay, severe intellectual disability, lack of speech, happy disposition, ataxia, epilepsy, and distinct behavioral profile [1,5,6,7]. This rare neurodevelopment disorder has a prevalence of 1 in 10,000–24,000 births [8].

AS has four molecular mechanisms of etiology: (1) deletion of the maternal copy of chromosome 15q11–q13 (del15q11–13, 70%) [9], (2) paternal uniparental disomy of chromosome 15q11–q13 (UPD, 2–7%) [10], (3) imprinting defects within chromosome 15q11–q13 that disrupt the expression of maternally inherited UBE3A (3–5%) [11], and (4) maternally inherited *UBE3A* mutations (10%) [12]. The clinical picture is variable in different genotypes. Generally, patients with a deletion type have a more severe phenotype, and those with UPD or imprinting defects have a less severe phenotype [7,13,14,15,16,17]. Genotype–phenotype correlation should be highlighted for accurate prediction and genetic consultation.

Children with AS usually have normal prenatal and birth history, normal metabolic and hematologic, and chemical laboratory results. The following four frequent clinical characteristics can be seen in all patients: severe developmental delay, movement or balance disorder, behavioral abnormality, and speech disorder (Table 1). Developmental delay often becomes evident by 6 months of age [18]. Movement or balance disorders usually include ataxia of gait and/or tremulous movement of the limbs. Most individuals lack speech completely, and few can speak a few words [19]. Receptive language is less impaired and is better than expressive language [14]. Frequent clinical characteristics, including microcephaly and seizures, occur in >80% of children with AS, often developing by 3 years of age [17]. A characteristic electroencephalogram (EEG) “signature” can also be found in 80% of children with AS [20,21], which may be an important hint for diagnosis [22]. Children with AS have several associated clinical features (Table 1). These children are easily excited and have an apparent happy demeanor associated with frequent laughter, hyperactivity, stereotypes, and proactive social contact [23,24]. Most children have sleep disturbance with reduced need for sleep and with long or frequent periods of wakefulness during the night [25,26,27,28,29].

## 2. Molecular Genetics and Diagnostics

**Figure 1:**
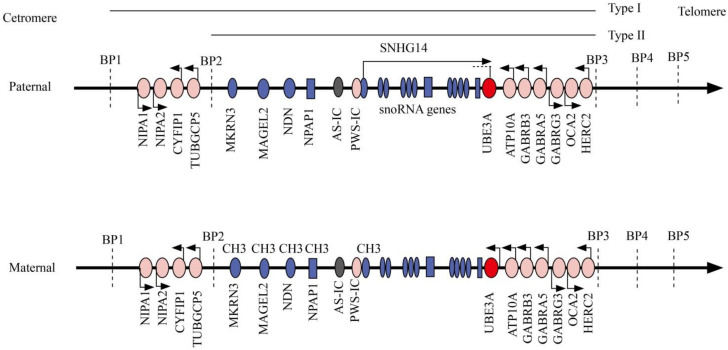
Human chromosome 15q11–13 region. Paternal and maternal chromosome 15q11–13 regions around the Angelman syndrome imprinting center (AS-IC) and Prader–Willi syndrome imprinting center (PWS-IC) are presented. Paternally expressed genes are indicated as deep blue, maternally expressed genes are indicated in red, and genes expressed from both parental alleles are indicated as pink. Transcription orientation is noted with arrows. Class I and class II deletions are indicated as horizontal lines. BP, breakpoint cluster region; CH3, methylation; snoRNA, small nucleolar RNA; *SNRPN*, small nuclear ribonucleoprotein-associated protein N; *UBE3A*, ubiquitin–protein ligase E3A.

Human chromosome 15q11–q13 contains a cluster of imprinted genes. In the imprinted region, methylation and gene expression are regulated by a bipartite imprinting center located in the small nuclear ribonucleoprotein associated protein N (SNRPN) region [31]. Genes in the imprinted center are clustered into paternally expressed only genes, maternally expressed only genes, and biallelically expressed genes (Figure 1). Differential expression of these maternal and paternal expression genes causes sister imprinting disorders: AS and Prader–Willi syndrome (PWS) [32].

*MKRN3, MAGEL2, NDN, PWRN1, NPAP1, SNURF-SNRPN,* and several C/D box small nucleolar RNA (snoRNA) genes are paternally expressed genes [33]. The CpG islands in the promoter regions are differentially imprinted: the paternal allele is unmethylated and is expressed, whereas the maternal allele is methylated and repressed [34,35]. Loss of expression of these paternally expressed only genes causes PWS.

The pathogenesis of AS is similar to PWS. *UBE3A* is a maternally expressed gene: the unmethylated maternal allele is expressed, and the methylated paternal allele is repressed. Imprinted expression of *UBE3A* is regulated by small nucleolar RNA host gene 14 (SNHG14) with a noncoding antisense transcript as the product which is started at the paternal SNRPN promoter [36] (Figure 1). In neuronal cells of normal individuals, the paternal region lacks methylation, and the paternally derived *UBE3A* gene is silenced [37]; the maternal region is methylated, SNHG14 is not expressed, resulting in *UBE3A* gene transcription [38,39]. Any genetic causes leading to a non-functional UBE3A protein will result in a knockout of neuronal UBE3A and lead to AS [40].

Lack of UBE3A protein expression in the brain of children with AS can lead to abnormal ubiquitination in Purkinje cells in the cerebellum [37,41,42]. Abnormal ubiquitination is attributed to the abnormality in the nigrostriatal pathway along with the cerebellum in animal models, which produce phenotypes that include motor impairments, synaptic plasticity, and repaired memory in AS [43,44,45]. Major aspects of the core clinical phenotypes of AS, including cognitive, language and motor deficits, are results of impaired long-range connection between the cerebellar and cortical networks [46].

**Figure 2:**
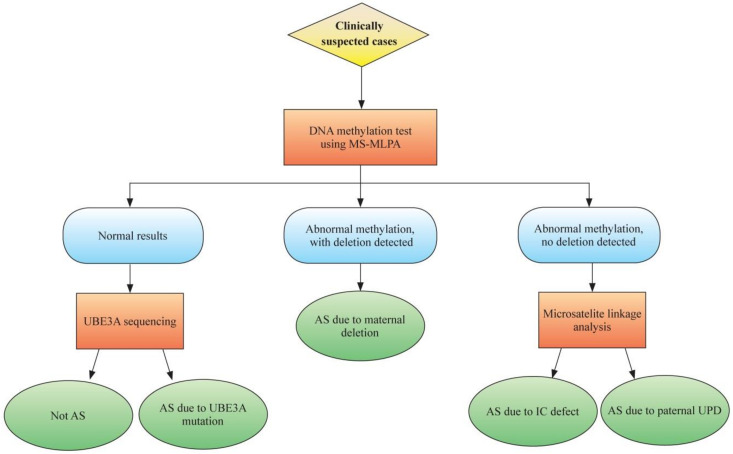
Molecular diagnostics for Angelman syndrome (AS). For those suspected with AS clinically, methylation analysis of the chromosome 15q11–13 region can be performed using methylation-sensitive multiplex ligation-dependent probe amplification (MS-MLPA). For those with normal methylation results, *UBE3A* gene sequencing is recommended to detect the *UBE3A* gene mutation. If *UBE3A* gene mutation is not detected, AS is excluded, and other diseases should be considered. For those with abnormal methylation results and deletion detected, AS due to del15q11–13 is diagnosed; for those without deletion, microsatellite linkage analysis can be done to differentiate paternal uniparental disomy (UPD) or imprinting defects.

Although the criteria for diagnosis of AS were defined in 2005 based on the common phenotypes of AS, a definitive diagnosis still depends on molecular testing. Differential methylation of chromosome 15q11–q13 provides the basis for molecular diagnosis (Figure 1). For those suspected with AS clinically, the first diagnostic modality is methylation analysis of the chromosome 15q11–13 region, methylation-sensitive multiplex ligation-dependent probe amplification (MS-MLPA). For those with normal methylation results, *UBE3A* gene sequencing is recommended to detect the *UBE3A* gene mutation. If *UBE3A* gene mutation is not detected, AS is excluded, and other diseases should be considered. For those with abnormal methylation results and deletion detected, AS due to del15q11–13 is diagnosed; for those without deletion, microsatellite linkage analysis can be done to differentiate UPD or imprinting defects (Figure 2).

It is necessary to differentiate several microdeletion syndromes and single-gene disorders that resemble AS. Chromosome microarray testing can be used to detect microdeletion syndromes, including Phelan–McDermid syndrome (22q13.3 deletion), MBD5 haploinsufficiency syndrome (2q23.1 deletion), and KANSL1 haploinsufficiency syndrome (17q21.31 deletion) [18,47]. If the results of microarray analysis are normal, single-gene disorders should be considered, which include Pitt–Hopkins syndrome (TCF4 haploinsufficiency), Christianson syndrome (*SLC9A6* mutation), Mowat–Wilson syndrome (ZEB2 haploinsufficiency), and Rett syndrome (*MECP2* mutations) [18].

Several pilot newborn screening programs have been undertaken for the early diagnosis of AS. Mahmoud et al. carried out a pilot newborn screening program and established a cost-effective newborn screening test for early diagnosis of PWS [48], which is also applicable to early diagnosis of AS. Ferreira et al. used a methylation-sensitive high-resolution melting method to analyze DNA extracted from dried blood spots. This method provides an accurate approach for genetic screening of imprinting-related disorders (PWS and AS) in newborns [49]. Early identification by newborn screening will not only achieve early efficient intervention but also will decrease costly medical evaluations.

Defining the exact molecular mechanism of AS is beneficial for genetic consultations. The risk of recurrence in parents of different genotypes is variable. In AS cases due to maternal deletion, the risk of recurrence is less than 1% [50]; for cases with paternal UPD, the risk of recurrence is less than 1/200; however, for those rare cases of structural defects of chromosome 15, including Robertsonian translocation, the risk is as high of 100%. For those due to IC defect, the risk of occurrence is 50% or 1% for those with or without deletion in maternal chromosome 15 [50]. For those with de novo *UBE3A* mutation, the risk of occurrence is near to 0; and for those with *UBE3A* mutation in both cases and their mothers, the risk is 50% [50]. Therefore, genetic analysis should be undertaken in the cases together with parents for genetic consultation.

Exploring the disease mechanism will also benefit targeted gene, and molecular therapies for AS. *UBE3A* critically impacts early brain development, and reactivation of *UBE3A* gene expression can prevent the onset of behavioral deficits [51]. Researchers are identifying novel therapeutics aimed at correcting pathophysiological deficits of AS and restoring the loss of UBE3A expression in the brain [52]. Restoring the function of UBE3A is the most promising therapeutic modality for AS.

## 3. Genotype–Phenotype Correlation in Angelman Syndrome

The severity of the phenotype depends on the molecular etiology. Individuals with a deletion commonly present with a more severe phenotype, whereas those with non-deletion have slightly milder and more variable presentations [7,53,54]. Intrauterine and postnatal growth, neurobehavioral and neuropsychiatric development phenotypes in children with AS depend on the genotype. Previous findings have revealed that patients with AS caused by del15q11–13 appear to have worse development, cognitive skills, albinism, ataxia, and more autistic features than individuals with other genotypes [13,53,55,56,57,58]. Children with *UBE3A* mutation appear to have less severe phenotypes with a nearly normal development quotient [58]. Below we review genotype–phenotype correlations based on different genotypes (Table 2).

### 3.1. AS Due to Maternal del15q11–13

Most patients with AS have maternal 15q11–13 deletions at a length of 5–6 Mb. The deletion is classified into two types based on the deletion length: Class I patients have breakpoints at BP1 and BP3 with various noncoding regions deleted (~6 Mb, ~16 genes), and Class II have breakpoints at BP2 and BP3 with a deletion of ~12 genes at a length of 5 Mb [8] (Figure 1). Classes I and II deletions represent 95% of AS patients with del15q11–13. Four evolutionarily conserved genes (*NIPA-1*, *NIPA-2*, *CYF1P1*, and *GCP5*) are located between BP1 and BP2, which are involved in central nervous system development and functioning [59]. Deletion of these genes may result in speech impairment and developmental delay. Genes causing the difference also include three GABAA receptor subunit genes (*GABRB3, GABRG3, GABRA5*) that are single-copies for the deletion genotypes but are intact for all non-deletion genotypes [8].

Developmental studies indicate that children with AS caused by a deletion are developmentally more delayed across all domains compared with those due to a *UBE3A* pathogenic variant or UPD [14,54,60]. Cognitive skills are much lower in the deletion group than in the non-deletion group [14,60]. Delayed gross and fine motor skills are more severe in the deletion group [14,53,60,61,62]. Seizure and microcephaly have been reported to be more common and severe in the deletion group [53,63,64,65]. The deletion subtype is associated with the most severe epilepsy phenotype; in contrast, non-deletion patients may have relatively late-onset seizures [64,66]. Mertz et al. found that children with a deletion type had significantly reduced developmental age regarding visual perception, receptive language, and expressive language compared with those having a *UBE3A* mutation and pUPD [58]. Therefore, children with deletion type had lower response rates to the social reinforcement paradigm than those with non-deletion type [67]; in other words, children with the deletion type are more difficult to treat using social intervention methods. Hypopigmentation was also significantly more prevalent in the deletion group [64].

For different types of deletion, children with Class I deletion are reported to have lower expressive and total language abilities than those having Class II deletion [55,57]. Children with BP1—BP3 deletion have more daily, disabling seizures, a higher frequency of seizures, and recurrent seizures aggravated by fever [16]. Class II deletion is associated with >50% intermittent rhythmic theta activity and normal posterior rhythm, whereas Class I deletion is associated with <50% intermittent rhythmic theta activity and epileptiform discharges during wakefulness [68]. Burnside et al. found that with more deleted genes and with locations nearer to the centromere, patients may have a higher prevalence of microcephaly, epilepsy, ataxia, speech disorders, and autistic symptoms [59]. The study results are conflicting on the difference of clinical severity in different deletion subtypes. Some studies found no difference in language abilities and cognitive function between Class I and Class II patients [8,13,14,56,58]. Additional studies should be carried out to delineate genotype–phenotype correlations of Class I and II deletion types.

### 3.2. AS Due to Paternal Uniparental Disomy for Chromosome15q11–q13

For children with paternal UPD of chromosome 15q11–q13, the structure and number of chromosomes are normal. However, there are paternally imprinted (maternal expressed) genes within the chromosomal region 15q11–q13, and maternally imprinted genes have been elevated but with very little expression of UBE3A. The developmental profiles are generally similar for patients due to UPD and imprinting defects. Both subtypes generally had slightly lower overall age equivalent scores and growth score equivalents and had slower rates of growth than the *UBE3A* mutation subtype across all domains. However, children with UPD or imprinting defects had higher overall age equivalent scores and growth score equivalents than those with both deletion classes [54]. Compared to the patients with deletion type, patients with paternal UPD have a much lower prevalence of epilepsy, better development and expressive language ability; some patients may even speak 2–7 words [58,59]. The UPD subtype is also associated with the lowest frequency of epilepsy and exhibits the least severe epilepsy phenotype [66]. Varela et al. found that swallowing disorders, hypotonia, and microcephaly in the UPD type are less severe than those in the deletion type [57].

Children with UPD presented significantly more severe hyperphagic behavior, hyperphagic severity, and hyperphagic drive than children in the other genetic groups [69]. These children also have significantly higher birth weight and birth length, being taller at five years of age than children with other genetic types [69]. Children with UPD and imprinting defects may have a higher risk of obesity than those with 15q11.2–q13 deletions and *UBE3A* mutations [15,70]. Sleep problems are more prevalent in children with UPD and *UBE3A* mutations compared with other types [64].

### 3.3. AS Due to Imprinting Defect

The imprinting control region 15q11–q13 cluster has been designated the “imprinting center” (IC). The IC can control the differential expression of alleles in varying tissues. The mechanism of the imprinting defect is related to failed imprinting or lacking submicroscopic structure [71]. Only maternal-of-origin-specific *UBE3A* genes can be found in neuronal cells. The maternally active *UBE3A* gene lacks differential DNA methylation. Therefore, no *UBE3A* gene is expressed in the brain of AS patients due to the imprinting defect. The phenotypes in patients with the imprinting defect are similar to those with UPD. They have better language and intellectual intelligence than the deletion type. Almost all patients with imprinting defect or UPD will become overweight before 44 months of age. The imprinting defect group may benefit more from behavioral interventions because they showed a high rate of reinforcement by social stimuli [67].

### 3.4. AS Due to Pathogenic UBE3A Mutation

Patients with *UBE3A* mutations have the mildest clinical presentations among the four genetic types. The developmental quotient is the highest among different types, and children due to *UBE3A* mutation have higher scores and greater rates of skill attainment in all development domains than other types [54]. These patients have relatively normal adaptive behaviors and are without obesity or overweight. Some patients can present symptoms of tremor in the head, limbs, and trunk [72]. However, attention should be paid to the severity of epilepsy in the *UBE3A* mutation subtype, which ranks second after the deletion subtype [64].

## 4. Conclusions

The current studies revealed that AS patients with different genetic types may have different phenotypes in performance, seizure, behavior, sleep, and other aspects. However, the genotype–phenotype correlation atlas has been drawn based on numerous studies with sample sizes varying in different genotypes. Many studies are limited owing to the small number of patients in a certain genotype, particularly the non-deletion types. Other genotype–phenotype correlations will be delineated if a larger number of patients are studied. Another limitation is that patients with AS from different regions have not been compared due to under-registration and underreporting. More data on carefully phenotyped patients from a global Angelman registry and multi-center study will benefit the development of genotype–phenotype correlations of AS.

Further studies should focus on several main issues, including epilepsy and neurodevelopmental outcomes that need further precise interventions. Understanding the pathophysiology of the different genotypes and the genotype–phenotype correlations will offer an opportunity for individualized treatment, genetic counseling, and better outcomes. Genotype–phenotype correlations based on larger data sets should be carried out to identify new treatment modalities, including gene therapy.

## Figures and Tables

**Table 1:** Clinical characteristics of Angelman syndrome (adapted from Williams et al. [30]).

Consistent (100%)	Frequent (>80%)	Associated (<80%)
Normal prenatal and birth history, no major birth defects Normal metabolic, hematologic, and chemical laboratory results Severe developmental delay Movement or balance disorder, usually ataxia of gait and/or tremulous movement of the limbs Behavioral uniqueness: frequent laughter/smiling; apparent happy demeanor; excitability, often with hand-flapping movements; hypermotoric behavior; short attention span Speech impairment: none or minimal use of words, non-verbal communication skills higher than verbal ones	Delayed or disproportionately slow growth in head circumference: usually resulting in absolute or relative microcephaly by age 2 years Epilepsy: usually starting before age 3 years Abnormal electroencephalogram (EEG): a characteristic pattern of large-amplitude slow-spike waves	Flat occiput; Occipital groove; Protruding tongue; Tongue thrusting; Suck/swallowing disorders; Feeding problems and/or muscle hypotonia during infancy; Prognathia; Wide mouth, wide-spaced teeth; Frequent drooling; excessive chewing/mouthing behaviors; Strabismus.

**Table 2:** Comparison of major phenotypes of different subtypes.

Major Aspects	AS Due to Maternal del15q11–13	AS Due to Non-Deletion		
		Paternal Uniparental Disomy for Chromosome15q11–q13 (UPD)	Imprinting Defect	Pathogenic UBE3A Mutation
Development	More delayed across all development domains than other types Cognitive skills lower than other types Delayed gross and fine motor skills more severe Reduced developmental age regarding visual perception, receptive language, and expressive language	Higher overall age equivalent scores and growth score equivalents than deleion type but lower than UBE3A mutation subtype; Better development and expressive language ability in patients with UPD and imprinting defect Higher scores and greater rates of skill attainment in all development domains in patients UBE3A mutation		
Seizures	More common and severe in the deletion group	Lower prevalence of epilepsy, and more with late-onset seizures. UPD subtype has the lowest frequency of epilepsy and exhibits the least severe epilepsy phenotype The severity of epilepsy in the UBE3A mutation subtype ranks second after the deletion subtype		
Behavior	Lower response rates to the social reinforcement paradigm than other types	The imprinting defect a high rate of reinforcement by social stimuli. Patients with UBE3A mutations		
Sleep	Common in all subtypes but Sleep problems are more prevalent in children with UPD and UBE3A mutations			
Others	Higher rate of hypopigmentation	UPD and imprinting defects have a higher risk of obesity than deletion type		

## Data Availability

No data are available for this review article.
